# Biomechanical and neural correlates of FastFES versus Fast gait training in individuals post stroke: a randomized control trial study protocol

**DOI:** 10.3389/fneur.2026.1792419

**Published:** 2026-07-02

**Authors:** Vyoma Parikh, Alexandra Slusarenko, Jacob Spencer, Jasmine M. Hope, Fisayo K. Aloba, Amit S. Grewal, Bennett L. Alterman, Keenan Batts, Catherine F. Mason, Laura Zajac-Cox, Sarah Caston, Michael R. Borich, Joe Nocera, Trisha M. Kesar

**Affiliations:** 1 Division of Physical Therapy, Department of Rehabilitation Medicine, Emory University School of Medicine, Atlanta, GA, United States; 2 Department of Physical Therapy, College of Allied Health Sciences, University of Illinois Chicago, Chicago, IL, United States; 3 School of Psychology, Georgia Institute of Technology, Atlanta, GA, United States; 4 Neuroscience Graduate Program, Emory University School of Medicine, Atlanta, GA, United States; 5 Emory University Hospital, Atlanta, GA, United States; 6 Department of Physical Therapy, College of Health Professions, Mercer University, Atlanta, GA, United States; 7 Center for Physical Therapy and Movement Science, Emory University School of Medicine, Atlanta, GA, United States; 8 Department of Neurology, Emory University School of Medicine, Atlanta, GA, United States

**Keywords:** Cerebrovascular accident (CVA), energy expenditure, exercise, functional electrical stimulation, Hoffman’s reflexes, peripheral nerve stimulation, stepping practice, transcranial magnetic stimulation

## Abstract

**Background:**

Fast gait training, individually and when combined with functional electrical stimulation (FastFES), has been shown to improve walking function in individuals post stroke. However, the neural mechanisms underlying the effects of these two gait training interventions are poorly understood. The purpose of this mechanism-focused gait rehabilitation randomized clinical trial is to assess the effects of Fast and FastFES gait training interventions on corticospinal neurophysiology, gait biomechanics, energy cost, and walking function in individuals with chronic post-stroke hemiparesis.

**Methods:**

In this randomized clinical trial, participants with chronic stroke are recruited and randomized to receive one of two gait training interventions—FastFES or Fast. Participants in each intervention group receive 12 sessions of gait training, with each training session comprising 30 min of training. During FastFES training, electrical stimulation is delivered to ankle dorsi- and plantar-flexor muscles during paretic swing phase and late stance phase, respectively. Evaluations of clinical, gait biomechanics, neurophysiological, and energy cost outcomes are performed at baseline, after completion of 12 training session (post12), and at 3-weeks and 6-weeks after completion of training (3-week follow up, 6-week follow up), to measure longitudinal effects of gait training. Additional evaluations are performed at completion of 3 and 6 training sessions (post3 and post6) to measure the time course of change during gait training. Upon completion of the study, planned analyses will include between-group comparisons of FastFES versus Fast gait training on training-induced changes in corticomotor and spinal excitability, gait biomechanics outcomes such as peak anterior ground reaction force, as well as association of training-induced changes in corticospinal neurophysiology and gait biomechanics with clinical and energy cost measures.

**Discussion:**

By elucidating the biomechanical and neural correlates underlying gait training-induced changes in locomotor function, this study promises to build on existing evidence supporting the clinical effects of FastFES and Fast gait training. The long-term goal of this study is to inform the development of neurobiology-informed, personalized, and innovative strategies to enhance the effectiveness of stroke gait rehabilitation.

**Clinical trial registration:**

clinicaltrials.gov, identifier NCT04380454.

## Background

Stroke is a leading cause of adult disability (1). Stroke induces a cascade of neurophysiologic changes in the cortical and spinal circuits (2–8) that result in biomechanical gait impairments (reduced paretic propulsion, footdrop) and gait dysfunction (reduced speed and endurance) (9–15). Stroke-related gait deficits are complex and multi-factorial, often persisting in over two-thirds of stroke survivors at discharge from rehabilitation (9–13, 16, 17). Post-stroke gait impairments negatively impact the kinematics and kinetics of all joints in the affected lower limb and all phases of the gait cycle, (10, 18–20), reducing community participation and quality of life (9–13, 16, 17). Enhancing the efficacy of stroke gait rehabilitation is an important goal for research and clinical practice (21–29). Rehabilitation-induced improvements in walking function rely on various factors such as biomechanics, energy expenditure, as well as the structure and function of corticospinal sensorimotor neural pathways. Increasing gait speed is a major focus of gait rehabilitation (30–35), however, there is a lack of consensus on the choice of intervention that maximizes gait speed while also promoting improved gait quality, energy cost, and neuromotor excitability (36–41).

Gait training interventions such as overground and treadmill walking at faster speeds have been shown to increase walking speed, but there is high inter-individual variability in training responses, often with only a subset of stroke participants showing clinically meaningful improvements in gait (responders) (32, 33, 36–43). Additionally, training-induced increases in gait speed may occur via diverse biomechanical mechanisms (34, 35, 44, 45) including compensatory actions by the nonparetic leg, despite poor paretic leg coordination (46). Furthermore, current neurorehabilitation philosophies based on neuroplasticity principles also strive for restitution of more normal gait patterns or gait quality to engage normal neuromotor pathways during walking (5, 47, 48).

The current mechanism-focused clinical study evaluates two clinically relevant gait interventions. Fast treadmill walking (Fast), our control intervention, is an evidence-based gait training intervention, comprising high-intensity, high-repetition, and bilateral stepping practice (49–53). Fast gait training provides repetitive stepping practice and aerobic exercise, which may encourage bilateral neuroplasticity (47, 54). However, without adjunctive feedback or cues (verbal, biofeedback, stimulation), Fast gait training is not targeted to specific gait deficits or the paretic leg. Importantly, neural correlates underlying Fast training are unclear based on previously published studies (55, 56). The second intervention being evaluated in our study, FastFES, provides unique advantages as a paradigm for probing biomechanics and neuroplasticity mechanisms of gait training. FastFES gait training combines Fast treadmill training and functional electrical stimulation (FES). FES is applied in a task-specific manner to the paretic limb ankle plantar- and dorsi-flexor muscles during the terminal stance and swing phases, respectively (42, 57–59). Unlike most gait interventions that include bilateral stepping practice, FastFES gait training targets the paretic leg ankle muscles by using intermittent FES to target both the ankle dorsiflexors and plantarflexors (60), aiming to promote motor learning of appropriate muscle activation patterns (61). Thus, FastFES gait training offers the ability to specifically target impairments in paretic propulsion and swing phase ankle kinematics (58, 62, 63) during high-intensity treadmill and over-ground stepping practice. From previously published work, FastFES gait training has been shown to improve gait function and energy cost (43, 58, 61, 63–66). In addition to enhancing the capacity to walk faster, reducing energy cost is crucial for sustaining faster gait speeds and perhaps promoting community activity (43). We hypothesize that in contrast to Fast gait training, FastFES gait training may promote greater use of the paretic leg for forward propulsion, thereby reducing inter-limb biomechanical asymmetry, which in turn reduces energy cost.

Experience-dependent neuroplasticity forms a fundamental process underlying training-induced behavioral improvements. However, our lack of understanding of neuroplasticity mechanisms underlying gait interventions continues to be a barrier to improving gait rehabilitation outcomes (5, 36–38, 40, 41, 67–70). Stroke leads to a decrease in M1 and corticospinal tract (CST) excitability (71) in the lesioned hemisphere, and elevated spinal reflex excitability (72, 73). Gait training may induce concurrent plasticity in multiple neural circuits (8, 74, 75). Using only gait function outcomes or a singular neurophysiologic outcome (e.g., transcranial magnetic stimulation (TMS) motor threshold) to measure training effects, will, therefore, likely be inadequate (32, 41, 76). Furthermore, FES capitalizes on state-dependence and task-specificity of plasticity-induction by delivering motor-level stimulation during gait superimposed with volitional activation (77, 78). During FES, activation of cutaneous and muscle-tendon receptors provide increased afferent input to corticospinal circuits (77, 78), which increases corticomotor excitability (79–81). Patterned stimulation also induces adaptive plasticity in spinal circuits (82), and may promote learning through enhanced spinocerebellar drive (78). Changes in TMS-evoked responses after conventional FES for footdrop have been explored, but previous investigations have either focused on short-term responses to FES or lacked rigorous controls (e.g., 6-month use of footdrop stimulator) (77, 83, 84). Furthermore, H-reflexes, when measured concurrently with TMS, can help identify the specific site and mechanisms of training-induced plasticity, while also providing insights into sensorimotor symptoms such as spasticity and excessive coactivation (72, 73). Thus, to address these gaps, this clinical trial aims to determine if and how FastFES and Fast training interventions modulate excitability of cortical and spinal neural circuits impacted by stroke and implicated in control of locomotion.

The overall purpose of this mechanism-focused randomized clinical trial protocol is to assess the biomechanical, neurophysiologic, and clinical effects of 12 sessions of Fast and FastFES gait training interventions in individuals with chronic post-stroke hemiparesis. Gait biomechanics, energy cost, cortical and spinal excitability, and clinical measures of walking function will be evaluated at *baseline*, *Post3*, *Post6*, *Post12*, *3-week and 6-week follow-ups*. Our specific aims are to evaluate the effects of FastFES and Fast on gait biomechanics asymmetry (Aim 1), corticospinal tract, M1, and spinal excitability (Aim 2), and associations between neurophysiologic versus gait biomechanics and clinical effects (Aim 3). We hypothesize that compared to Fast training, FastFES gait training will result in: (1) larger increases in gait symmetry, with an increase in forward propulsion of the paretic versus non-paretic leg, (2) greater reduction in energy cost, and (3) greater modulation of lesioned corticospinal tract and spinal reflex excitability.

## Methods

### Overall study design

**Figure 1:**
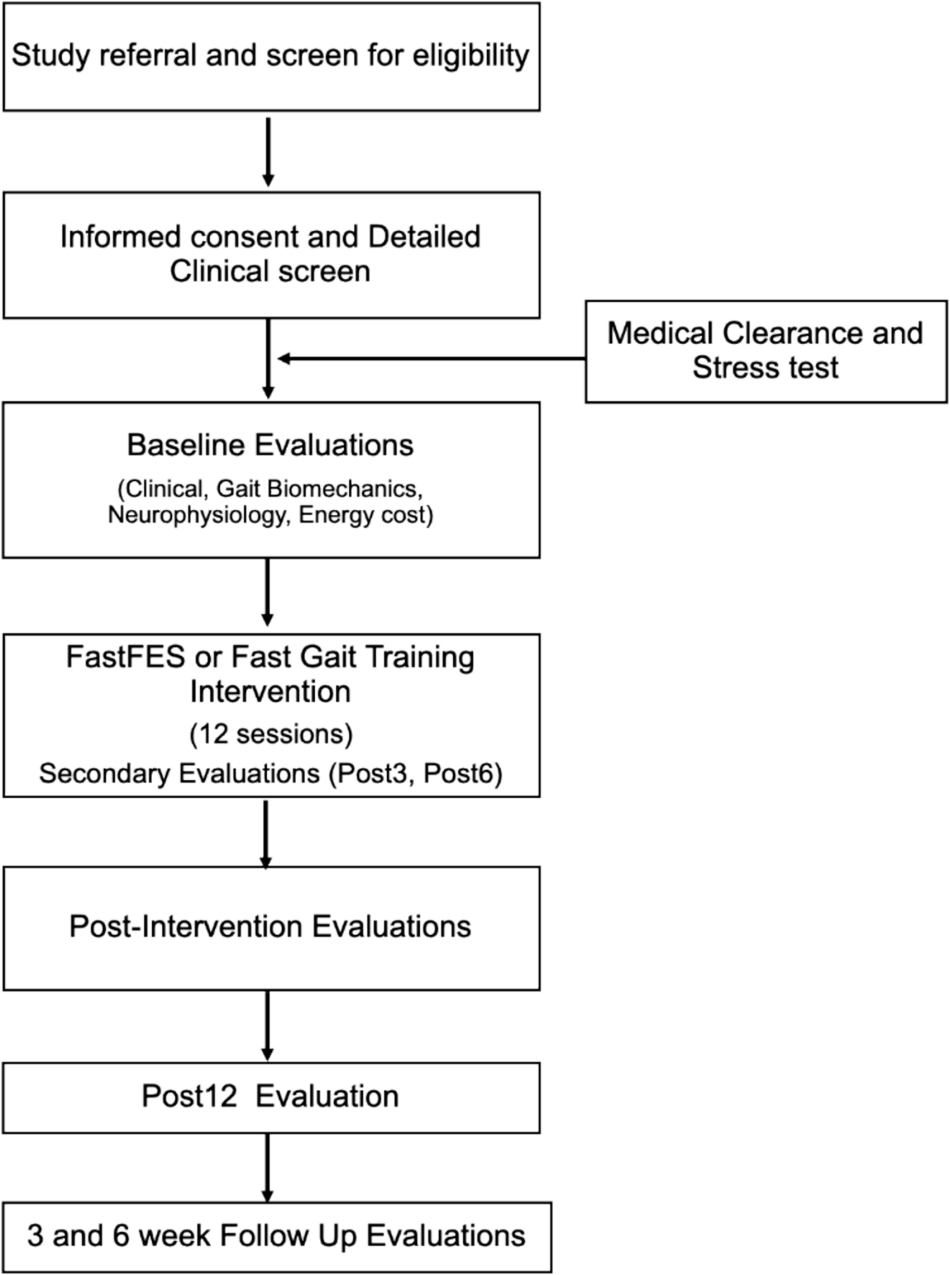
Study flowchart. Overview of the study protocol for gait training intervention in individuals with stroke. Primary timepoints for the study are post 12 evaluation, 3 and 6-week follow up evaluations. Secondary timepoints include post 3 and post 6 session evaluation.

This study is a mechanism-focused randomized controlled clinical trial evaluating the effects of FastFES and Fast gait training intervention protocols on individuals with chronic post-stroke hemiparesis (>6 months post stroke) (Figure 1). Participants are randomized to one of the two gait training intervention groups. The participants and the therapist providing gait training were not being blinded to group allocation. However, research staff and physical therapist completing clinical assessments are blinded to group allocation.

### Screening, consent, and confirming study eligibility

#### Study enrollment

Individuals with chronic stroke (> 6 months post-stroke) over the age of 35 years are recruited from the greater Atlanta area and university-related clinics. All participants provide written informed consent, and the study protocol is approved by the institutional review board at Emory University. The participants’ eligibility for study participation is confirmed via an initial phone screen, medical records review, clinical evaluation, and other baseline evaluations. We also obtain written medical clearance for study participation before the participants can be randomized or start the gait training interventions.

#### Participant inclusion and exclusion criteria

*Inclusion criteria*: at least 6 months since stroke, age 35–90 years, cortical or subcortical ischemic stroke, ability to walk 10 meters with or without assistive device, medical clearance by a cardiologist indicating sufficient cardiovascular health to participate in the assessments and interventions, sufficient walking function and ankle stability to walk on treadmill for 2 min at self-selected speed without an orthosis, and resting heart rate 40–100 bpm.

*Exclusion Criteria*: hemorrhagic stroke, signs of cerebellar involvement (ataxic gait or decreased coordination), inability to communicate with investigators, neglect/hemianopia, unexplained dizziness in past 6 months, musculoskeletal conditions limiting walking, neurologic diagnoses other than stroke, contraindications to TMS (85).

These inclusion/exclusion criteria are based on previously published FastFES gait training studies (42, 61, 63, 86), ongoing studies from our lab, as well as TMS safety guidelines. To account for attrition rate due to failed eligibility criteria or dropout, a total of 65 individuals may be recruited.

#### Accessing and collecting personal health information

Initial screening, chart review, clinical evaluations, gait evaluations, and evaluations of cortical and spinal physiology are used to capture subject data. All subject data are de-identified and compiled. De-identified data may be stored for future use. De-identified data may be disseminated through group discussions, presentations, and publications. Data are stored in an electronic, secure, and internally hosted web-based application designed exclusively to support data capture for research studies and facilitate compliance with institution’s HIPAA policies and procedures.

#### Informed consent

Screening and verbal consent is obtained by the PI and study team members via phone screening to evaluate eligibility for study. Written informed consent is obtained from participants in person using a paper form or using an electronic format via REDCap (87). In-person or electronic consent is obtained at the initial study visit and before any study-related procedures are initiated. During the informed consent session, participants are provided with detailed information about the study objectives, procedures, time commitment, and possible risks.

#### Procedures to confirm study eligibility

##### Medical clearance, exercise stress test, and MRI

Before starting the gait training intervention phase, participants must also receive a written medical clearance from their physician for study participation. Additionally, before the intervention phase, participants are referred for an exercise stress test (conducted under the supervision of a cardiologist) to confirm cardiovascular tolerance for intensive treadmill training. Participants may then also be referred for an MRI (magnetic resonance imaging) scan, used to obtain lesion location, neuro-navigation to guide transcranial magnetic stimulation (TMS) delivery, and future ancillary analyses.

##### Clinical evaluation

After confirming study eligibility, a physical therapist (PT) conducts a clinical evaluation comprising a battery of tests to assess gait and dynamic balance function, lower limb sensorimotor impairments, as well as other tests such as modified Ashworth spasticity score (88), and Stroke Impact Scale (89). Additionally, we determine their self-selected and fast treadmill speeds during a baseline biomechanical evaluation on the treadmill.

### Randomization to gait training intervention group

Intervention assignments for enrolled participants are stratified by baseline self-selected gait speed (obtained using during the 10-meter walk test at baseline clinical evaluation) of > or ≤ 0.6 m/s (90). Group assignments were generated in REDCap based on an algorithm setup by the study statistician using a pseudo-random number generator, with stratification based on baseline overground gait speed, with randomly permuted blocks. The study research coordinator runs this randomization step for each participant after they have completed study screening and pre-randomization procedures. Study staff and investigators are blinded to the randomization process and do not influence this step. Speed based stratification based on a baseline gait speed of ≥0.6 m/s was implemented to balance the Fast and FastFES groups by baseline walking function. The 0.6 m/s threshold helps ensure a similar distribution of lower- and higher-functioning walkers across groups, and to enable our study sample to include both household and community ambulators (90). We posit that speed-based stratification would facilitate sufficient variability in the study participant walking function while also enabling potential secondary subgroup analysis, in case gait speed may influence response to gait training (42). The clinical evaluators are blinded to treatment assignments.

### Study timepoints

#### Baseline evaluations

Before training, baseline evaluations are conducted over a 1-3-week period (Figure 1). The baselines involve 3–4 visits or sessions conducted on different weekdays. Baseline evaluations include: (i) clinical evaluation, (ii) gait biomechanics evaluation, (iii) neurophysiology evaluation, and (iv) energy cost of walking evaluation. Baseline neurophysiology data are measured over 1 or 2 sessions, and double baseline neurophysiology data are obtained if feasible.

#### Post-training and follow-up evaluations

Using the same procedures as baseline evaluations, post-training evaluations are conducted after 12 sessions of training (*Post12*) (Figure 1). Follow-up evaluations are conducted at 3- *(3-week Follow-up)* and 6-weeks *(6-week Follow-up)* after completion of gait training. Additional evaluations (biomechanics, neurophysiology, clinical) are conducted after 3 and 6 sessions of training (*Post3, Post6*) to evaluate short-term, training-induced changes as a predictor of long-term change. We try to complete the *Post12* evaluations within 1–10 days of completion of the last gait training session. Our goal is to try to complete the follow-up evaluations in the 3- and 6-week periods following completion of the last gait training session, with some variability due to scheduling constraints, holidays, or other pragmatic factors.

To promote participant retention, the study coordinator and study team utilize timely reminders via phone, text, or email related to upcoming training and evaluation sessions. The study can cover cost of transportation and/or parking, and the honorarium includes a small bonus for completing all study procedures. The study team records information related to participants who discontinue or deviate from intervention protocols, and the data collected to date would still be utilized for analysis, as appropriate (Table 1).

**Table 1:** Participant timeline: Schedule of enrollment, interventions, and assessments.

Timepoint	Trial period						
	Enrollment		Post-randomization			Close-out	
	Screening	Baseline	Post3 session	Post6 Session	Post12 Session	3-week follow up	6-week follow up
Enrollment							
Eligibility screen	X						
Informed consent	X						
MRI scan, medical clearance, exercise stress test	X						
Randomization		X					
Intervention							
FastFES gait training		X		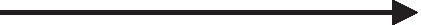	X		
Fast gait training		X		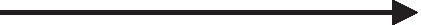	X		
Assessments							
10 meter walk test	X	X	X	X	X	X	X
6-min walk test		X	X	X	X	X	X
Timed up and go test		X	X	X	X	X	X
Clinical battery (FMA-LE, FGA, modified ashworth)		X			X		X
Stroke impact scale		X			X		X
Gait biomechanics		X	X	X	X	X	X
Neurophysiology		X	X	X	X	X	X
Energy cost (VO_2_)		X			X		X

*FMA-LE, Fugl Meyer Assessment Lower extremity test; FGA, Functional Gait Assessment.

### Gait training intervention phase

#### Methodology for evaluations

- Clinical evaluation

Clinical evaluation of walking function is performed by a licensed physical therapist (PT). Clinical testing comprises standard clinical tests of lower limb function. Clinical tests that measure International Classification of Function (ICF) domains of impairment include lower extremity proprioception through passive joint movement, light touch and protective sensation utilizing Semmes-Weinstein monofilaments, spasticity using the Modified Ashworth Scale (88), and lower limb motor function using the lower extremity portion of the Fugl-Meyer Assessment (91). Clinical tests that measure the ICF domain of activity include: (1) over ground self-selected and fast walking speed (10-meter walk test) (92, 93); (2) over-ground walking endurance measured by the distance ambulated during the 6-min walk test (94, 95); (3) assessment of gait and dynamic balance using the functional gait assessment (96), and the Timed Up and Go (TUG) test (93). Clinical tests under the ICF participation domain include the Stroke Impact Scale (89), Walk-12 questionnaire (97), and Activity-specific Balance Confidence Scale (98).

In addition, the participant’s fast walking speed is determined as the fastest speed they can maintain safely during a 2-min bout of treadmill walking. For some subjects, clinical testing may only comprise a subset of the outcomes listed above. All the clinical outcomes are measured at the study primary timepoints (baseline, *post12*, and *6-week follow-up*). Additionally, at *Post3* and *Post6*, an abbreviated set of clinical outcomes (overground gait speed, TUG, 6-min walk test) are obtained.

- Gait biomechanics evaluations

To set up for gait analysis, reflective markers are attached to the participant’s lower extremities (57, 99). Elastic wraps (Fabrifoam, USA) are secured around the thighs, calves and pelvis onto which small, thermoplastic shells containing reflective markers are attached. Additional markers are taped to the participant’s shoes and on the upper back, shoulder, hip, knee, and ankle joints with adhesive skin tape. Marker data is collected using a 7-camera motion analyses system (Vicon, Oxford, UK). During treadmill walking, ground reaction forces are collected using a treadmill instrumented with two 6-component force platforms under each belt (Bertec, USA). Motion analysis data are collected during a 1-s-long static standing calibration trial. Next, 15- to 60-s-long dynamic gait trials are recorded as subjects walk on a treadmill at their self-selected and fast speeds. During treadmill walking, the force platforms embedded within the treadmill are used to record ground reaction force data. During treadmill walking, subjects wear a ceiling-mounted safety harness without body weight support and can hold on to a front handrail. Subjects are allowed rest breaks as often as requested. Similar to the procedures described for the training sessions, subjects’ heart rates, perceived exertion, and blood pressure are monitored during the session. Biomechanical assessments (gait kinematics and kinetics) are collected at *Baseline*, *Post3*, *Post6*, *Post12*, *3-week follow-up*, and at *6-week follow-up*.

- Neurophysiology evaluations

*Procedures for measurement of corticospinal tract excitability*: Corticospinal excitability is assessed using transcranial magnetic stimulation (TMS). TMS is delivered to the hotspot of the paretic soleus muscle over the lesioned hemisphere using a custom batwing coil connected to a Magstim 200^2^ stimulator (Magstim Co., Wales, UK). The hotspot of the paretic soleus is identified as the location on the scalp that elicits the largest amplitude motor-evoked potentials (MEPs) at the lowest intensity (100). Surface electromyography (EMG) sensors are attached on the skin overlying the paretic Soleus and Tibialis Anterior (TA) muscle bellies. To promote consistency across time points, electrode placement was performed by the same investigators whenever possible, using palpation of anatomical landmarks and active muscle contraction to identify the ground and recording electrode sites for the TA and soleus muscles, with placement completed by a licensed rehabilitation clinician or an investigator experienced in EMG setup. Stereotactic neuro-navigation is used to maintain accurate coil positioning within and across sessions. During neurophysiologic evaluations, to reduce variability in background EMG, participant posture, positions of lower and upper limb joints and background activation state of bilateral ankle muscles (soleus and tibialis anterior) are monitored throughout the session. In addition, to account for the effects of background EMG on neurophysiology data, during processing, we will visually assess MEP and H-reflex data to ensure EMG activity prior to and after MEP trace. Additionally, if needed, we will be able to normalize the data to background EMG or Mmax to further limit the effect of background activity on MEP amplitude. TMS data are collected in a quiet standing position (101, 102). Baseline assessment is used to determine the most appropriate test position (quiet standing, sit active, sit rest) for testing. In cases of increased movement, imbalance, safety concerns, or fatigability noted during quiet standing during testing, if needed, the testing condition is switched to a sit active posture.

If a participant is unable to maintain a quiet standing position, TMS data are collected in seated at rest (sit rest) or sitting while maintaining a low-level contraction of the target ankle muscles (sit active), depending on their tolerance level. If being tested in the sit active position, participants are provided visual feedback of ongoing soleus EMG to maintain background activity at 10% maximal voluntary contraction (MVC) activation level. Maintaining consistent background EMG helps stabilize the motor output at the motoneuron level (100, 103, 104) and reduces inter-trial and inter-session variability (105). Active motor threshold (aMT) is determined using a computerized algorithm (106) as the TMS intensity that elicits MEPs of amplitude >100uV for at least half of the pulses sent (85). TMS-evoked MEP amplitudes at 130% aMT are obtained by averaging peak-to-peak amplitudes of 10 to 20 TMS-evoked soleus MEPs.

Secondary outcomes of cortical excitability are measured using paired-pulse TMS. Paired-pulse data are collected to determine the magnitude of intracortical inhibition and facilitation of the primary motor cortex bilaterally. TMS is delivered using two Magstim 200^2^ stimulators connected via a BiStim^2^ module (Magstim Co., Wales, UK). Conditioning pulses are delivered at a range of intensities (70–130% of the active motor threshold determined for the target muscle). Both facilitatory (interstimulus interval of 12 ms) and inhibitory (interstimulus intervals or 2 ms and 100 ms) paired-pulse assessments are performed. Comparing mean peak-to-peak MEP amplitudes of the conditioned and non-conditioned stimuli are used to index short intracortical inhibition and facilitation (107).

*Procedures for assessment of spinal excitability*: Spinal excitability is assessed using peripheral electrical stimulation delivered to the posterior tibial nerve, innervating the paretic soleus muscle. Testing is completed with the participants in a quiet standing position. A self-adhesive 2-inch x 2-inch electrode is used as the anode which is placed over skin above the anterior aspect of the patella. A 1-inch round electrode is used as a cathode in the popliteal fossa to determine the hotspot that provides the best EMG response (108–110), EMG activity is recorded while 50–60 electrical stimuli (1 ms pulse duration, ranging in intensity from below H-reflex (H-max) threshold to above maximal M-wave (M-max) intensity), at least 5 s apart, are delivered over the posterior tibial nerve in the popliteal fossa (110, 111). Resulting recruitment curves are used to compute the maximal H-reflex and M-max, and the H-max/M-max ratio. Neurophysiological assessments are performed at *Baseline*, *Post3, Post6*, *Post12*, *3 week follow-up*, and *6 week follow-up*.

- Energy cost of walking evaluation (VO_2_ Testing)

During the energy cost evaluation, participants are asked to wear a fitted mask connected to a metabolic cart (COSMED Quark CPET, COSMED USA Inc., Concord, CA). In addition, participants wear a standard 12-lead ECG to monitor the participant’s heart rate activity. Once set up is complete, participants stand for 2 min to obtain baseline measurements. Participants then warm-up and familiarize themselves with the treadmill at half the speed of their self-selected treadmill speed for 2 min. Following warm up and treadmill familiarization, the participant completes 4 min of walking at their SS speed, followed by a 2-min recovery period in quiet standing. Breath-by-breath data during the entire evaluation are collected and primary outcomes will include distance walked, respiratory rate (RR; breaths·min^−1^), pulmonary ventilation (VE; L·min^−1^), oxygen uptake relative to body weight (VO_2_; ml·kg^−1^·min^−1^), expired carbon dioxide relative to body weight (VCO_2_; ml·kg^−1^·min^−1^), end-tidal partial pressure of carbon dioxide (P_ET_CO_2_), ventilatory equivalents of oxygen (VE·VO_2_^−1^), ventilatory equivalents for carbon dioxide (VE·VCO_2_^−1^), and respiratory exchange ratio (RER). Gross oxygen cost is determined using steady-state oxygen consumption data during the treadmill energy cost test divided by walking speed (relative VO_2_ cost per body weight per meter walked; ml·kg^−1^·m^−1^). Net oxygen cost is calculated by subtracting the resting oxygen cost from the walking oxygen cost (112–114). V0_2_ assessments are performed at *Baseline*, *Post12*, and *6 week follow-up* (112–114).

#### Methodology for gait training interventions

**Figure 2:**
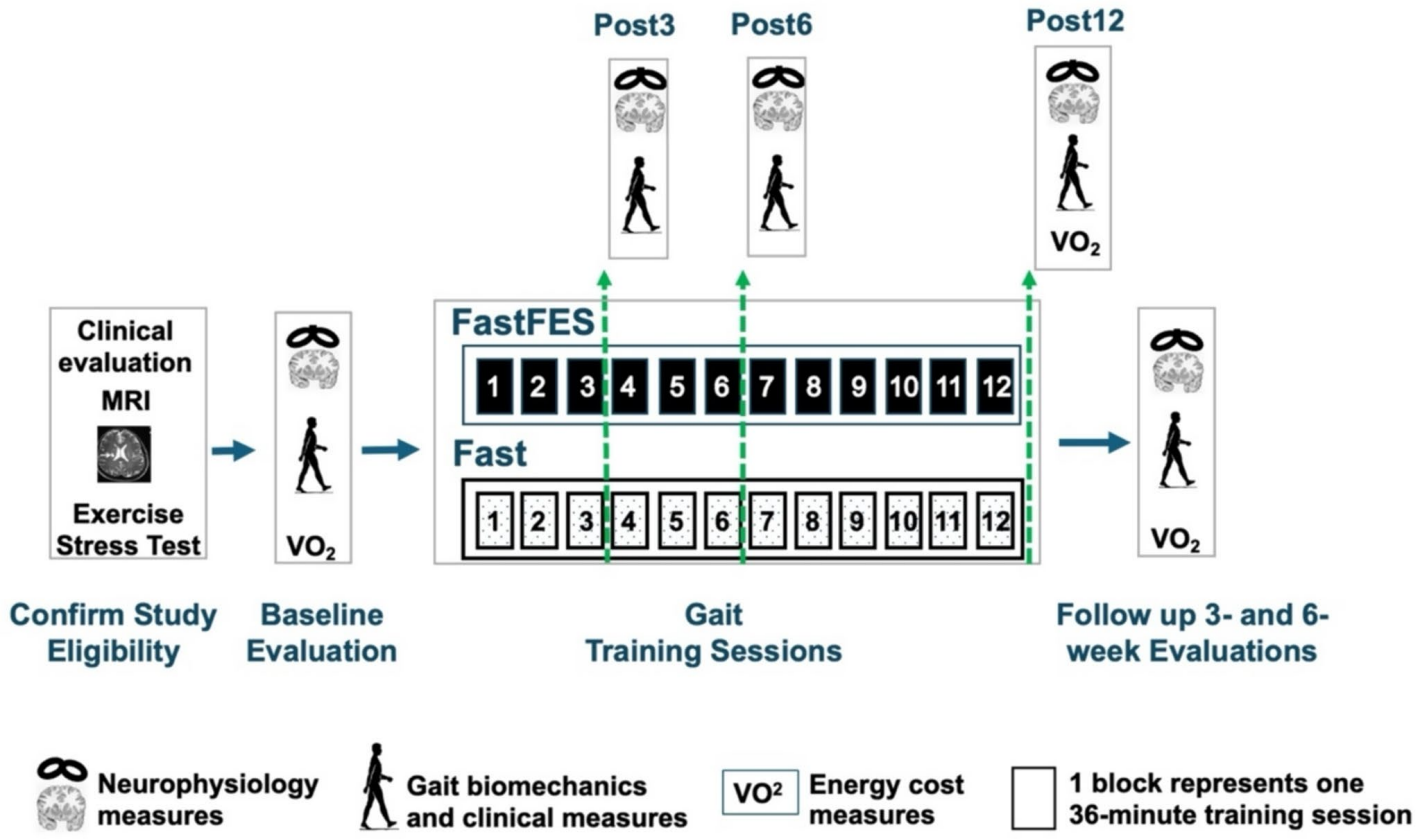
Schematic of overall study design. Baseline and post evaluations include measurement of gait biomechanics, clinical measures, cortical and spinal neurophysiology, and energy cost outcomes.

**Figure 3:**
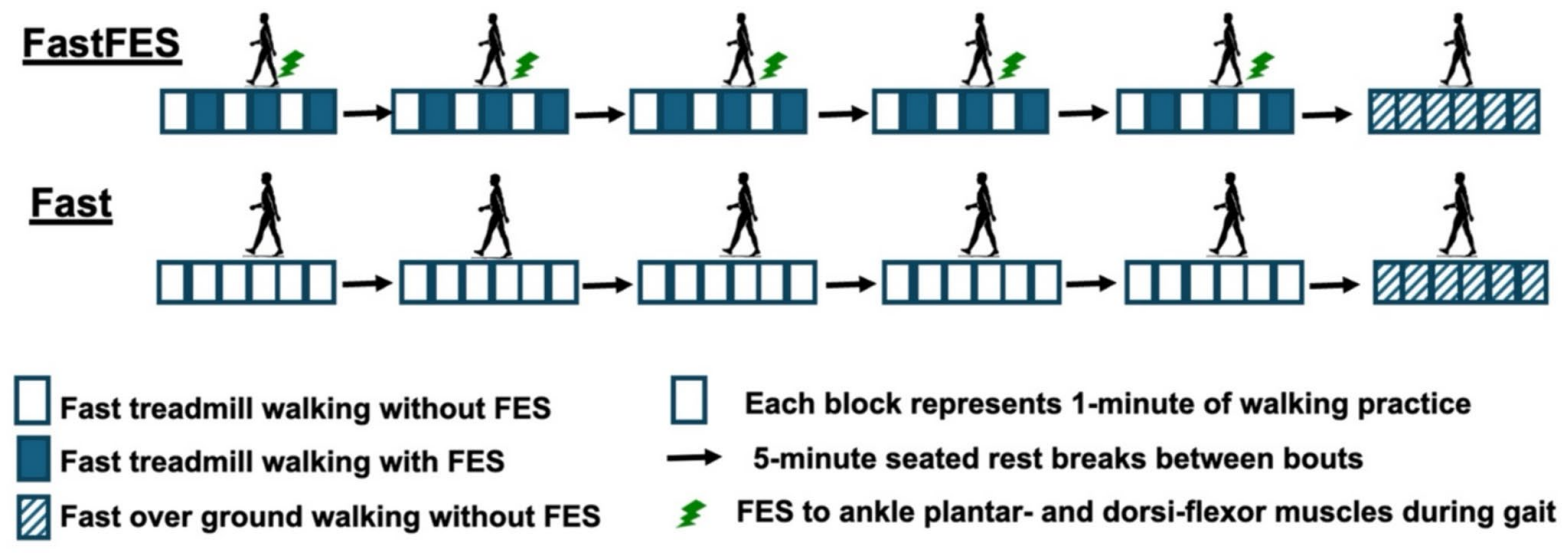
Methodology for FastFES and fast gait training sessions. Overview of methods for each session of FastFES and fast gait training intervention.

Participants are randomized to receive one of two gait training interventions: Fast training or FastFES. The two walking training interventions (fast treadmill walking with or without electrical stimulation) are well-matched in terms of duration, structure, intensity, and dosage of training (Figure 2). Both training interventions are comprised of 12 training sessions (2–3 sessions/week), with each training session consisting of a total of six 6-min walking bouts with approximately 5-min breaks between bouts (total stepping practice of 30 min per session) (Figure 2). The first 5 bouts comprise treadmill walking, and the last training bout (bout 6) may comprise 6-min of overground walking, during which subjects are tasked to walk as far as possible at a brisk pace while maintaining safety, demonstrating in-session carryover of mechanics learned during the preceding treadmill training bouts (Figure 3).

For safety, a PT guards the participants during both treadmill and over-ground walking bouts, with baseline walking aids utilized during over-ground bouts, if present. Gait training speed is determined and progressed to maintain moderate exercise intensity similar to previous studies (43, 115), and to be consistent with exercise guidelines (116, 117). The initial training speed is selected as a speed faster than the baseline self-selected speed and is determined in an individual-specific manner based on the PT’s judgement of stability and quality, while accounting for the participant’s ability to complete a 6-min bout safely at that speed. Both within and across gait training sessions, the training speed is progressed in an individual-specific manner based on exercise intensity heart rate, Rating of Perceived Exertion (RPE), biomechanical challenge, safety (evaluated by PT’s judgement), and participant’s tolerance or agreement (118). During each training session, the treadmill training speeds, RPE, and heart rate are recorded for each bout to enable quantification of training intensity (42, 43, 63, 86, 115).

*FES methodology:* An electrical stimulator is used to deliver stimulation during walking (Grass S8800 stimulator with SIU8TB stimulus isolation unit; UDel stimulator). A customized, real-time system (57, 99, 119, 120) (CompactRIO, National Instruments, TX) controls the stimulator and delivers stimulation during appropriate phases of the gait cycle. During FastFES, surface stimulation pads are attached to ankle dorsiflexors and plantarflexor muscles, and FES intensity is determined at the start of every training session. For the dorsiflexors and plantarflexors, the stimulation electrodes (bipolar setup) are positioned over the muscle belly of the tibialis anterior and gastrocnemius-soleus muscle bellies, respectively. The FES parameters used are similar to previously published work on the FastFES protocol (frequency: 30-Hz variable frequency trains; waveform: square wave; train duration: determined based on the gait cycle events detected by footswitches; pulse duration: 60 μs (99, 120, 121). The FES intensity for dorsiflexor and plantarflexors is determined for each individual and each training session as motor-level stimulation that elicits appropriate functional movements (adequate dorsiflexion in a seated position for dorsiflexor FES, lifting of heel off the floor in double support standing for plantarflexor FES) (115, 120). During FastFES treadmill training bouts, using footswitches and a custom program, ankle dorsiflexors and plantarflexors are stimulated (alternating 1-min periods) through the duration of the paretic swing phase and at the point of terminal stance phase of gait, respectively.

During the gait training sessions, subjects wear an overhead harness (no body-weight support) for safety. In addition, if needed, the subjects are allowed to hold on to the handrails during walking. Heart rate will be monitored throughout the session with a heart rate sensor that is placed on the chest under clothing (Polar USA, Lake Success, NY). Participants are asked to rate their perceived exertion on the Borg Scale of Perceived Exertion every 2 min throughout each 6-min walking bout. If the rate of perceived exertion exceeds 18 or blood pressure exceeds 170/100 mmHg, the session is stopped. Participants are provided rest breaks as needed (118). Similar stopping criteria may also be used for other sessions involving walking, such as energy cost testing, clinical evaluations, etc.

Scripted verbal instructions are provided during training to provide feedback to the participants to assist in understanding the goals of training, as well as to enable replication and generalizability of study results. For FastFES gait training, scripted instructions are provided during each training session to focus on the specific aspects of gait that the FES assists with (push off during late stance, dorsiflexion during swing phase), and to instruct participants to use intermittent 1-min periods without FES to learn to voluntarily activate their muscles appropriately during walking practice, encouraging carryover of FES-assisted mechanics. For Fast training, scripted instructions emphasize the importance of high-intensity, high-repetition stepping, with no specific instructions to focus on the paretic leg or ankle. A PT is present during all gait training sessions.

##### Dependent variables

- Biomechanical and gait performance variables

*Primary Measure:* Propulsive asymmetry between the non-paretic and paretic peak anterior ground reaction forces (AGRF) (122), which has been shown to correlate with walking function (123).

*Secondary Measures:* Energy cost (EC) of walking, which is calculated as the oxygen consumption during V0_2_ assessment collected during treadmill walking at SS speed, normalized to body weight (kg) and speed (m/min) to yield the energy cost (ml O^2^/kg/m) (43).

*Exploratory Measures:* Peak ankle plantarflexor moment, peak ankle power, circumduction (124), and pelvic excursion (20) during gait, which are obtained using inverse dynamics from gait biomechanical evaluation during two 30-s pre and post gait trials on the treadmill at self-selected and fast speeds.

- Clinical variables

Primary Measure: Self-selected and Fast Walking Speeds (10-Meter Walk Test).

*Secondary Measures*: Distance ambulated during the 6-Minute Walk Test and time to complete the Timed Up and Go (TUG) Test.

- Neurophysiological measures

*Primary Measure: Paretic soleus* TMS motor evoked potential (MEP) amplitude.

*Secondary Measures:* *Paretic soleus* intracortical facilitation (ICF) and H-max/M-max ratio.

##### Data monitoring

The study team (PI and designated co-I) will be responsible for data safety and monitoring all study procedures. The PI will oversee the organization and management of the study data on Redcap, paper copies of clinical report forms (e.g., for clinical outcomes), procedural reliability of gait biomechanics, neurophysiology, and clinical outcome data, as well as gait training methods. The PI will record any adverse events associated with the study, as stipulated by the institutional review board, and any reportable events will be documented and reported to the IRB.

##### Data management

Study demographic and clinical data will be stored in an electronic database system REDCap (Research Electronic Data Capture), which is a web-based application that provides a secure method to manage and support data for research studies. REDCap is compliant with the United States healthcare confidentiality legislation requirements. REDCap provides an ability to enter data easily and allows easy import and export of the data as needed for statistical analyses or audit purposes. Data entry and data verification will be completed by the study coordinator and research team member trained in REDCap processes. All physical data files and results are stored in a HIPAA-compliant manner. De-identified gait biomechanics, energy cost, and neurophysiology data will be stored on secure password-protected computers or servers.

Strategies to improve and monitor adherence to intervention will be used such as keeping track of the number of training sessions attended by participants using case report forms, notes, or REDCap, sending reminders to participants prior to scheduled sessions, and careful coordination of the post-training and follow-up study evaluations. Participants will be provided with instructions about any concomitant care that is prohibited during the trial, such as beginning any new rehabilitation program, or any medical or surgical interventions that may affect/interact with the study protocol. A quality improvement review or audit of the study protocol and documentation will be performed by the institution. Additionally, an annual review of the protocol and experimental sessions will be conducted by the PI and the study team.

### Statistical analysis

#### Sample size estimation, participation accrual, and missing data

Estimation of sample size was performed using data from previously collected preliminary data, which provided estimated effect sizes for training-induced change in gait propulsion symmetry (Aim 1) and MEP amplitude (Aim 2), and a power analysis was performed to achieve sufficient power to reject a null hypothesis of equal mean change compared to the population mean difference in change. Assuming a two-sided, two-sample unequal-variance t-test with a significance level *α* of 0.05, a cohort of n = 30 per group provides 87% power to detect a between-group difference in mean propulsion change of 15% (SD_Fast_ = 22.3; SD_FastFES_ = 13.4) and 96% power for a between-group difference in mean MEP change of 30.9% (SD_Fast_ = 27.5; SD_FastFes_ = 35.6). While Aim 3 is exploratory, the feasibility of our model-based approach is supported by prior regression analyses in similar stroke cohorts (n = 27 to 50) that successfully predicted therapy-induced gait speed changes using clinical and neuroimaging variables (42, 125). Standard data cleaning and identification of missing data will be completed. If missing data are present, further investigation will be conducted regarding the missing data and probable underlying causative factors or impact to study results. Our protocol and analysis plan to address and minimize missing data include rigorous screening and recruitment plans, retention plans, and an intention-to-treat framework. Additional plans to address missing data include sensitivity analyses to encompass different scenarios of assumptions and discuss consistency or discrepancy among them. The model-based means estimated from the mixed effects general linear model are unbiased with unbalanced and missing data, so long as missing data are missing at random. We do not expect to see informative censoring in the data. Should there be evidence that missing data are informative and related to dropout, more sophisticated statistical methods will be applied (e.g., random-coefficient selection models and random-coefficient pattern mixture models).

*Statistical Analysis for Aims 1 and 2.* Repeated-measures analysis of each dependent variable (e.g., propulsive asymmetry) to be performed with a means model via SAS MIXED Procedure (SAS Institute, Cary, NC), providing separate estimates of the means by time (baseline 1 and 2; *Post3, Post12; 3-week,* and *6-week follow-up*) and intervention arm (Fast or FastFES). The model will include 3 predictors (intervention, training time (categorical), and intervention-time interaction). A compound-symmetric variance–covariance form in repeated measurements will be assumed and robust estimates of standard errors of parameters will be used to perform statistical tests and construct 95% confidence intervals. A *p*-value ≤0.05 will be considered statistically significant.

*Statistical Analyses for Aim 3*. Our outcome (predicted) variables will be training-induced change in gait biomechani**c**s (gait asymmetry), energy cost, and gait function (speed, endurance) at *Post12* and *6-week follow-up*. Independent variables will be the baseline values and short-term training-induced change (at *Post3*) for paretic soleus MEP and H-max/M-max. We may use data points from *baseline* to *Post12* to estimate MEP slope, to determine if MEP is associated with gait symmetry improvement based on a 2-stage model. In stage 1, MEP slopes and intercepts will be estimated using a mixed-effects model specifying that MEP measures from *baseline* to *post12* follow a linear regression over time with a random slope and intercept for each patient. In stage 2, multiple linear-regression models will be fit for gait symmetry, estimated as slope from *Baseline* to *Post12* as outcome, with patient-specific slope and intercept estimates obtained from mixed-effects model as continuous covariates. Bootstrap bagging will identify stable and reliable predictors of outcome. Additional exploratory analyses may be performed. We may conduct a re-specified sub-group analysis (126) to examine subgroups based on baseline speed, by including interaction between intervention and subgroup. We can also evaluate the time course of change with training to study rate of improvement (during training) and decay (during follow-ups).

##### Adverse event monitoring and protection of human subjects from harm

Any incidental or secondary study findings that are deemed relevant or that affect the study risks or benefits are monitored by the study team. Relevant adverse events, if any, are reported to the institutional review board as per the recommended IRB guidelines. Changes in informed consent and protocol documents are made to reflect any modifications made to the study protocol as well as any incidental findings, as needed.

Some of the possible risks of the study are loss of balance or falls during gait training or clinical evaluations, muscle soreness or excessive muscle fatigue from gait training with or without FES, risk of skin irritation or burns due to stimulation malfunction, and headache or discomfort from neurophysiological assessments. To mitigate the above risks, all gait training interventions occur with a PT present, participants are screened to ensure they do not have any contraindications to TMS, electrical stimulation, or walking exercise, and participants’ exertion, heart rate, and overall wellness are monitored during the evaluation and gait training sessions. Rest breaks are also built into evaluation and training sessions to reduce fatigue and muscle soreness.

##### Dissemination

We plan to submit the findings from this study to be published in peer-reviewed journals and present them at local and national conferences. The Principal Investigator will conduct regular meetings with the research team to update the dissemination strategy and review authorship policies. The Principal Investigator will also prepare and distribute a summary of the research results that are accessible and understandable to the general public, based on requests from participants.

## Discussion

The current study represents an initial effort toward gaining a more comprehensive understanding of the changes in walking function, gait biomechanics, and cortical and spinal excitability induced by post-stroke gait rehabilitation interventions in the same cohort of stroke survivors. Although the emphasis on increasing gait speed is a major goal of stroke gait rehabilitation, it is also important to consider the underlying biomechanical and neural mechanisms to align with the emerging standards of precision medicine, which represent the future of clinical rehabilitation. Building upon prior published evidence regarding the effects of the FastFES gait training intervention, this study seeks to elucidate, for the first time, the neural processes underlying Fast and FastFES, as well as to evaluate how training-induced changes in corticomotor excitability associate with concomitant changes in gait biomechanics and energy cost in individuals following stroke.

Stroke leads to neurophysiologic changes in cortical and spinal circuits (2–8) that result in biomechanical gait impairments (reduced paretic propulsion, footdrop) and gait dysfunction (reduced speed and endurance) (1, 9–14). The current mechanism-focused clinical trial takes a first step toward validating theoretical frameworks regarding neurobiological (top-down) and biomechanics (bottom-up) mechanisms of Fast and FastFES gait treatments. We hypothesize that FastFES is a *targeted* intervention. FES delivered at appropriate phases of the gait cycle provides motor level stimulation-induced cues to improve ankle propulsion. FES is delivered only to the paretic ankle muscles, enhancing afferent ascending drive as well as descending corticomotor drive preferentially to *lesioned* CST, M1, and spinal circuits (which will be evaluated using neurophysiologic outcomes). Increased corticomotor drive in lesioned corticomotor circuits in turn may promote improved timing and intensity of muscle activation in the paretic plantar- and dorsi-flexor muscles, increasing plantarflexor moment and propulsion from the *paretic* ankle, and improving inter-limb gait symmetry (which will be evaluated using our biomechanical outcomes). Additionally, due to promotion of more symmetrical gait biomechanics, FastFES may reduce energy cost of walking, as previously supported by Awad et al. (43) (which will be evaluated using our energy cost outcome). In contrast, Fast may be a *non-targeted* intervention that provides similar structure, dose, and intensity of stepping practice as FastFES, but does not include stimulation, and no specific instructions are provided to target practice to the paretic leg or specific deficits at the paretic ankle. We predict that Fast, therefore, should not preferentially increase corticomotor output in the *lesioned* CST. Fast should also not induce preferential increases in the *paretic* plantarflexor moment or propulsion, and thus may not promote gait symmetry. After our results test these hypotheses, the long-term impact of this work will be to develop methods to match individual neurobiological, physiological, biomechanical, and clinical characteristics to gait treatments that can maximally restore both gait speed and gait quality.

By understanding the neural and biomechanical mechanisms associated with FastFES gait training, the findings of this study can provide critical insights regarding *why* and *for whom* FastFES and Fast might be most effective. We will conduct a longitudinal assessment comparing the effects of FES gait training and Fast gait training on corticomotor excitability, with cortical and spinal sensorimotor circuit neurophysiology, gait biomechanics, and key clinical functional outcomes measured at multiple timepoints before, after, and during gait training. Additionally, we aim to investigate how training-induced neural plasticity relates to improvements in gait performance, gait biomechanics, and energy expenditure. Finally, our data may also elucidate the role of baseline clinical and neurophysiological characteristics in identifying ‘responders’ to the gait training intervention.

Another key goal of our study is to determine whether the neuromotor processes underlying FastFES result in not only a greater magnitude of but also longer-lasting improvements in post-stroke gait compared to Fast gait training, by utilizing the 3- and 6-week follow-up data. Additionally, we can explore how changes in gait asymmetry relate to energy expenditure (VO₂) following both Fast and FastFES interventions. A better understanding of the neural mechanisms and dose–response time courses underlying gait training can aid with the future development of individualized and innovative intervention strategies and dosing regimens to improve the efficacy of gait rehabilitation.

We would like to acknowledge several limitations of our study. We did not set any participant exclusion criteria based on participant gait speed or baseline sensorimotor impairments with the rationale that heterogeneity in baseline walking ability is an important consideration for interpreting intervention response and having generalizable intervention results. Another limitation of this protocol is its multi-aim, multi-domain design, which includes distinct primary hypotheses for each aim rather than a single overarching primary variable for the trial. The planned analyses involve multiple comparisons across outcomes and time points without formal adjustment for multiplicity, inducing risk of type I error, and findings beyond the primary outcome specified for each aim should therefore be interpreted cautiously as exploratory. While we do not have plans to statistically adjust for multiplicity of outcome variables, such analyses can be addressed more fully in future studies designed to confirm and extend the current study findings. In addition, because the study enrolls ambulatory individuals with chronic stroke, generalizability may be greatest for relatively higher-functioning chronic stroke populations. In addition, because of the longitudinal nature of the trial, electrode placement sites will not be marked on the skin across sessions, which may introduce some variability in repeated neurophysiological measurements despite efforts to standardize electrode placement procedures.

### Trial status

This clinical trial is registered at ClinicalTrials.gov (Trial Registration number: NCT04380454). The trial was registered on 4th May 2020. IRB approvals from Emory University were obtained on 29th February 2019. This protocol paper reflects the study protocol created in 2019, and any amendments to the protocol have been tracked through the clinicaltrials.gov web-based system. Recruitment began on 16th March 2021. The total study duration, including enrollment and analysis, is estimated to be 6 years. Recruitment was completed on 31st August 2025.
